# Autophagy-Induced HDAC6 Activity During Hypoxia Regulates Mitochondrial Energy Metabolism Through the β-Catenin/COUP-TFII Axis in Hepatocellular Carcinoma Cells

**DOI:** 10.3389/fonc.2021.742460

**Published:** 2021-11-11

**Authors:** Xiaoyu Yan, Xianzhi Qu, Buhan Liu, Yuanxin Zhao, Long Xu, Sihang Yu, Jian Wang, Liying Wang, Jing Su

**Affiliations:** ^1^ Key Laboratory of Pathobiology, Ministry of Education, Department of Pathophysiology, College of Basic Medical Sciences, Jilin University, Changchun, China; ^2^ Department of Hepatobiliary & Pancreatic Surgery, The Second Hospital of Jilin University, Jilin University, Changchun, China; ^3^ Department of Molecular Biology, College of Basic Medical Sciences, Norman Bethune Health Science Center, Jilin University, Changchun, China

**Keywords:** hypoxia, HDAC6, β-catenin, hepatocellular carcinoma, autophagy, mitochondrial energy metabolism

## Abstract

Hypoxia is one of the main driving forces that results in poor outcomes and drug resistance in hepatocellular carcinoma (HCC). As the critical cellular oxygen sensor, mitochondria respond to hypoxic stress by sending retrograde signals to the nucleus that initiate adaptive metabolic responses and maintain the survival of HCC cells. Increasing evidence suggested autophagy contributes to sustain mitochondrial metabolic and quality control. Understanding how mitochondria communicate with the nucleus and alter transcription may provide promising targets for HCC treatment. In this study, we found mitochondrial undergoes selective degradation by autophagy under hypoxia. Furthermore, autophagy-activated HDAC6 not only promoted the nuclear translocation of β-catenin but also increased the affinity of β-catenin to the transcription repressor chicken ovalbumin upstream promoter-transcription factor 2 (COUP-TF II), which suppressed mitochondrial oxidative phosphorylation-related genes transcription. Our data showed that autophagy served as a critical mediator of integrating mitochondrial energy metabolism and nuclear transcription. HDAC6 may be a potential target for reducing the survival of HCC cells by interrupting mitochondria-nucleus crosstalk.

## Introduction

Hepatocellular carcinoma (HCC) is one of the most malignant tumor types and the third leading cause of cancer-related death worldwide (1). Unfortunately, the 5-year overall survival rate of HCC patients is less than 20% (2). Hypoxia is known to be an important factor in the HCC tumor microenvironment that is caused by rapid tumor growth and a dysfunctional liver microvascular system. Recent studies have suggested that hypoxia-induced remodeling of mitochondrial metabolism is responsible for HCC recurrence and poor outcomes after chemotherapy (3, 4).

Mitochondria are not only the major cellular oxygen consumer but also the critical energy factory of the cell. They monitor the cellular metabolic status and integrate signaling pathways to maintain the cellular energy supply. Under hypoxic conditions, mitochondrial oxidative phosphorylation (OXPHOS) is suppressed, which is beneficial for cell survival (5, 6). Recent studies have demonstrated that dysregulated and damaged mitochondria are removed by autophagy in a process termed mitophagy that is critical for mitochondrial quality control. During this process, mitochondria are encircled by double-membrane vesicles and delivered to lysosomes for degradation (7). Interestingly, autophagy is also responsible for critical responses during tumorigenesis. Previous studies in HCC have found that inhibiting mitophagy promoted p53 phosphorylation and nuclear translocation in hepatic cancer stem cells (8). These studies indicated that dysregulated mitochondria-induced mitophagy may result in nuclear transcriptional responses. Thus, communication between mitochondria and the nucleus controls metabolic homeostasis during stress, such as that from hypoxia.

HDAC6 is a member of the class IIb histone deacetylase (KDACs) family. Recent studies have shown that HDAC6 controls mitochondrial respiration by interacting with the mitochondrial protein PHB2 (9). Importantly, HDAC6 also interacts with cortactin, through which it plays pivotal roles in autophagosome-lysosome fusion during autophagic clearance (10). Lee et al. demonstrated that HDAC6 was required for Parkin-mediated mitophagy (11). These data clarified its role in aggresome-autophagy and the acetylation modification of cytoplasmic proteins. Additionally, selective inhibitors of HDAC6, such as ACY-241 and ACY-1215, have become promising candidates for anticancer therapy (12). Thus, further investigations into whether HDAC6 participates in remodeling energy metabolism in HCC during hypoxia and how it functions in the coordination of mitochondria-nucleus crosstalk are needed.

There is a working hypothesis that pro-survival signaling from the Wnt/β-catenin pathway is frequently hyperactivated in HCC (13). β-catenin is a core transcriptional activator that translocates to the nucleus, where it recruits co-activators and transcription factors such as the nuclear DNA-binding proteins T cell factor/lymphoid enhancer-binding factor (TCF/LEF), HIF1α, and forkhead box protein O (FOXO)1 to regulate target gene expression (14–16). Although HDAC6 has been shown to regulate β-catenin stability, more recent studies have suggested that acetylation modifications at different sites may affect the affinity of β-catenin for different co-factors (17, 18).

In this study, we further explored the role of autophagy-induced HDAC6 activity in mitochondrial energy metabolism and nuclear transcription in HCC. Our findings suggested the activated HDAC6 promoted the nuclear translocation of β-catenin by suppressing its acetylation under hypoxia. Deacetylated β-catenin could recruit the transcription repressor COUP-TFII, which inhibited the transcription of OXPHOS-related genes, resulting in mitochondrial suppression during hypoxia in HCC cells. These results suggested that HDAC6 could be a messenger for the crosstalk between mitochondria and nucleus. Thus, targeting HDAC6 may be a potential therapeutic method against hypoxia-induced HCC cell survival.

## Materials and Methods

### Reagents and Antibodies

3-(4, 5-Dimethylthiazol-2-yl)-2, 5-diphenyltetrazolium bromide (MTT) were purchased from Sigma-Aldrich (St. Louis, MO, USA). Tubacin (HY-13428) was purchased from Med Chem Express (Monmouth Junction, NJ). ACY-1215 was purchased from Selleck (shanghai, China). The following anti-body were used: anti-MPC1, anti-NR2F2 (Abcam, Cambridge, MA, USA); anti-HDAC6, anti-HIF1α, anti-β-Catenin, anti-LaminA/C, anti-beta actin (Proteintech, Chicago, IL, USA); anti-Acetyl-β-Catenin (Lys49) #9534, anti-acetyl-α-Tubulin (Lys40) #3971 (Cell Signaling Technology, Beverly, Massachusetts, USA).

### Cell Culture and Transfection

SNU-387 and SNU-449 cells were obtained from American Type Culture Collection (ATCC; Rockville, MD, USA). Both cell lines were cultured in DMEM culture medium (Gibco Life Technologies, Carlsbad, CA, USA) supplemented with 10% fetal bovine serum (Invitrogen, Carlsbad, CA, USA) at 37°C in a 5% CO_2_. Hypoxia (1% O_2_, 5% CO_2_, 95% N_2_) was achieved using a Hypo-Hyper Oxygen System. Lipofectamine 3000 reagent (Invitrogen) was used for transfection. The pcDNA3.1-wt-β-Catenin, pcDNA3.1-β-Catenin-K49Q, pcDNA3.1-β-Catenin-K49R and empty vector (NC) were constructed by Sangon Biotech (Shanghai, China).

### Cell Viability Assays

The Real-Time Cell Analyzer (RTCA, Roche/ACEA Biosciences) was used for detecting cell proliferation, which monitored cell adhesion and proliferation through sensing electrical impedance. 8× 10^3^ cells were seeded in E-plate 16 (ACEA Biosciences) with 37°C under normoxia and hypoxia. The cell index was measured every 5 min for 24 h and the increasing slop was used for cell growth rate measurement.

### Transmission Electron Microscopy

Cells were fixed at 4°C in 2% glutaraldehyde. Then, they were post fixed in 2% OsO4 and the 50-nm thin sections were stained with 4% uranyl acetate and 2.5% lead nitrate for electron microscopy analysis.

### Oxygen Consumption Rate Measurement

5×10^4^ cells were seeded in 96-well plates. 1% O_2_ and Tubacin were used for indicated wells. After loading oxygen-sensitive time-fluorescent probes (Mito-Xpress, Luxcel Bioscience, Cork, Ireland), the oxygen consumption rate was measured using Omega luminometer (BMG Labtech, Ortenberg, Germany).

### Mito-Tracker Green Staining

Centrifuge to obtain a cell pellet and aspirate the supernatant. Resuspend the cells gently in prewarmed (37°C) staining solution containing the mito-tracker probe for 15 minutes under growth conditions. After staining is complete, re-pellet the cells by centrifugation and resuspend cells in fresh prewarmed medium or buffer. Cells were analyzed by Accuri C6 Flow Cytometry (BD Biosciences, Franklin Lakes, NJ, USA).

### Flow Cytometry for Cell Cycle

Cells (30 × 104 cells/well) were seeded in 6-well plates. After exposure to different treatment, cells incubated in propidium iodide/Triton X-100 staining solution with RNase A for 10 min at room temperature. Samples were analyzed by Accuri C6 Flow Cytometry (BD Biosciences, Franklin Lakes, NJ, USA). And its cell cycle-dependent distribution was analyzed using the ModFit LT 3.0 software.

### ATP Production Analysis

Cells were treated with Tubacin for 12 h with 1% oxygen. ATP production was determined using an ATP Bioluminescence Assay Kit (Beyotime Technology). Omega luminometer was used to measure the values (BMG Labtech, Ortenberg, Germany).

### Chromatin Immunoprecipitation

Cells treated with 1% oxygen and Simple ChIP plus enzymatic chromatin IP kit (magnetic beads) #9005 (Cell Signaling Technology, Beverly, Massachusetts, USA) was used as described in manufacturer’s instructions. Briefly, cells were fixed with formaldehyde to cross-link histone and non-histone proteins to DNA. The chromatin was digested with micrococcal nuclease into 150-900 bp DNA/protein fragments. Anti-β-Catenin antibodies was added followed by incubation overnight at 4°C and was captured by protein G magnetic beads. The protein/DNA complex was disintegrated, and DNA was purified for analysis. High-throughput sequencing was performed by Shanghai Outdo Biotech Co., Ltd.

### High-Throughput Sequencing

For ChIP-seq data, sequencing reads were mapped to human reference genome hg19 assembly using Bowtie2. MACS peak calling module was applied to call peaks for β-Catenin by modeling the length of the protein-binding sequence. The number of paired-end reads that map to the sequence determined the relative abundance of the peek corresponding to the sequence. The threshold for peak calling was diffScore >= 50 which was exactly P value<=1e-5 [diffScore= -10*LOG10 (pvalue)]. Differential peaks between the samples were identified by MAnorm then annotated. In addition, hypergeometric optimization of motif enrichment (HOMER) was employed for the transcription factor motif enrichment calculation on selected peaks. Peak signals flanking transcription start sites were scaled and normalized to draw the density plot for the comparison between samples.

### Luciferase Assay

The cells were transfected with the TOP-FLASH (TOP) and HIF1α luciferase reporter plasmid using transfection reagent. After 1% O_2_ treatment, cells were collected and lysed. The luciferase activity was measured using the Dual-Luciferase assay kits (Promega, Madison, WI, USA) according to the manufacturer’s instructions.

### Protein Extraction and Western Blot Analysis

HCC cells were treated with a nuclear protein extraction kit (Beyotime Biotechnology, Wuhan, China) for nucleus extraction. Minute™ Mitochondrial Isolation Kit for Mammalian Cells (Invent Biotechnologies Inc., MN, USA). And Proteins were subjected to Western blotting as previously described (19).

### Immunoprecipitation Assay

Cells were lysed in NP40 lysis buffer. Measure the total protein amount by BCA assay. And lysates of 0.5 ml contained a total of 1 mg protein. Add 2 μg of primary antibody to the whole lysate and set up a negative control experiment with control IgG. Gently rock the incubations at 4°C overnight. After adding 50 μl Protein G sepharose bead (Beyotime, China) slurry to capture the immune-complex, gently rocked the mixture at 4°C for 4 h. Wash the beads 3–4 times with 1 ml 1× TBST and resuspend the pellet with SDS sample buffer. Heat at 95°C for 5 min and centrifuge at 10,000g. Then load supernatants onto an SDS-PAGE gel for western blotting analysis.

### Immunofluorescence Staining

Cells were washed and fixed in 4% (w/v) paraformaldehyde/PBS for 20 min. After permeabilized with 0.1% Triton X-100 for 15 min, bovine serum albumen was used for blocking. Then cells were incubated with primary antibody overnight at 4°C. FITC/Texas Red-conjugated secondary antibodies (1:200 dilution; Proteintech, Chicago, IL, USA) was used for cell staining. Images were acquired by an Olympus microscope system.

### RNA Extraction and Quantitative Real-Time PCR

Total RNA extracted and First-strand cDNAs synthesized were described as before. Quantitative real-time PCR was done by using the MX300P instrument (Agilent, USA) followed by a 3-step PCR protocol. The primers sequences for MPC1, 5’-ACTATGTCCGAAGCAAGGATTTC-3’, 5’-CGCCCACTGA TAATCTCTGGAG-3’, SDHA, 5’-CAAACAGGAACCCGAGGTTTT-3’, 5’-CAGCTTGGTAACACATGCTGTAT-3’, SDHB, 5’-ACAGCTCCCCGTATCAAGAAA-3’, 5’-GCATGATCTTCGGAAGGTCAA-3’, NDUFS1, 5’-TTAGCAAATCACCCATTGGACTG-3’, 5’-CCCCTCTAAAAATCGGCTCCTA-3’. The relative expression was calculated by ΔCt among different experimental groups normalized to ACTB expression

### HDAC6 Enzymatic Activity Measurement

Cells were seeded in 6-well plates and incubated with 1% O_2_. After centrifugalization, cells were re-suspended into PBS (PH=7.2-7.4). Sonication was used to break up membranes and release cell component. Centrifuged at the speed of 3000 rpm/min for 20 minutes and collected supernatant. The HDAC6 activity assay kits (Meimian, China) were used as described in the manufacturer’s instructions. In brief, testing and standard samples were added to micro-elisa stripplate and incubated for 30 min at 37°C. Wash the wells and add HRP-conjugate regent in each sample. The enzymatic activity was analyzed at 450 nm after adding chromogenic agen.

### *In Vivo* Xenograft Experiments

All experimental procedures were performed in accordance with the National Institutes of Health guide for the care and use of laboratory animals. The study was approved by Institutional Animal Research Committee of China Medical University. SNU-387 cells were subcutaneously injected into the nude mice (BALB/c, SPF grade, 18-20 g, 6 weeks old, and male) from the animal experimental center (Beijing, China). Mice were randomized in to two groups and intraperitoneally administered 50mg/kg ACY-1215 every two days for 2 weeks. The body weight and tumor volumes were measured. The expression of proteins and mRNA were evaluated.

### Statistical Analysis

All data were conducted using means ± SD and carried out using the Student’s t-tests. Differences were considered statistically significant for P values <0.05.

## Results

### Hypoxia Promoted HCC Cell Survival and Autophagy on Mitochondria

**Figure 1:**
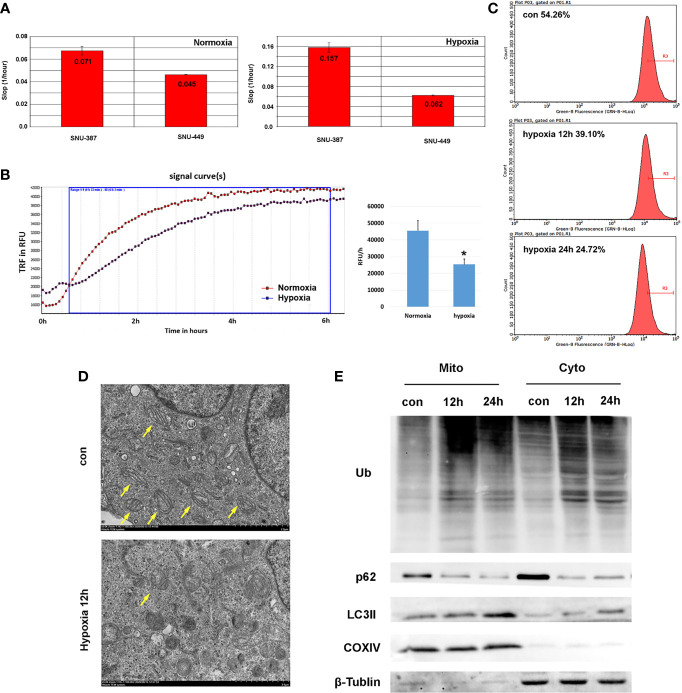
Hypoxia suppressed mitochondrial function and promoted mitophagy of HCC cells. **(A)** Cell proliferation rate was measured by RTCA system and the increasing slop was used for cell growth rate detection. **(B)** Measurement of OCR in SNU-387 cells exposed to hypoxia or normoxia for 6h (means ± SD, n = 3, *P < 0.05). **(C)** Mitochondrial mass was analysis by mito-tracker green in SNU-449 cells under hypoxia. **(D)** The morphology of SUN-387 cells mitochondria was observed with TEM under hypoxia. **(E)** Mitochondria of SNU-387 cells was isolated and the protein expression of autophagy were investigated.

Our results found that, compared with normoxia (21% O_2_), HCC cells showed increased viability with 1% oxygen treatment, which indicated that HCC cells may overcome normal barriers to proliferation, such as hypoxia, as they adapted to the ever-changing hostile tumor microenvironment (**Figure 1A**). Mitochondrial respiration was measured by the oxygen consumption rate (OCR), which indicated that 1% oxygen treatment limited the OXPHOS levels of HCC cells (**Figure 1B**).We assumed hypoxia may switch tumor cells from using OXPHOS to glycolysis as their primary energy source. Furthermore, we found that mitochondrial mass was reduced with prolonged hypoxia exposure (**Figure 1C**). Mitochondrial morphology changes rapidly in response to external stress and insults and is intimately linked to OXPHOS activity. Transmission electron microscopy (TEM) showed that hypoxia caused mitochondrial swelling and disorganized cristae. Importantly, we also observed increased numbers of autophagosomes under hypoxia (**Figure 1D**). To examine whether hypoxia promoted mitophagy in HCC cells, we further isolated mitochondria and investigated the levels of autophagy-related protein markers. The results showed the ubiquitinated protein was obviously accumulated on mitochondria. Furthermore, compared with cytoplasm, increasing LC3 was recruited to mitochondria under hypoxia; additionally, the key autophagy adaptor protein p62 was degraded significantly (**Figure 1E**). These studied suggested that hypoxia may cause mitochondria inhibition and induced autophagy on mitochondria in HCC cells.

### Autophagy-Induced HDAC6 Activity Participated in Mitochondrial Suppression in Hypoxic HCC Cells

**Figure 2:**
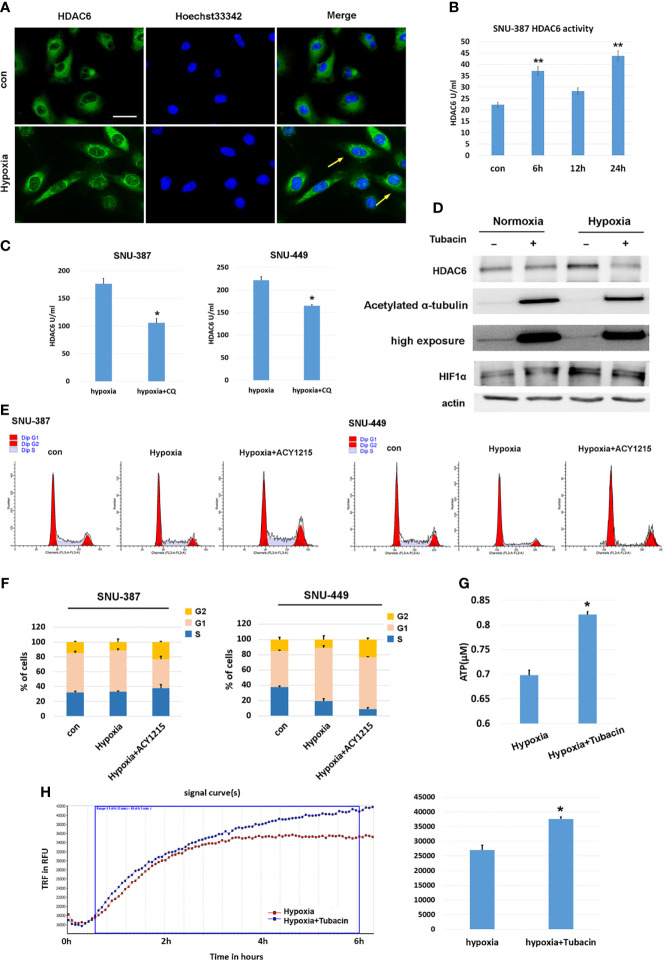
HDAC6 inhibition increased mitochondrial respiration and suppressed hypoxia-induced proliferation features of HCC cells. **(A)** Immunofluorescence staining for HDAC6 in SNU-449 cells under normoxic or hypoxic conditions. Scale bar = 60 μm. **(B)** The activity of HDAC6 was measured in SNU-387 cells (means ± SD, n = 3, **P < 0.01). **(C)** SNU-387 and SNU-449 cells were treated with CQ and the activity of HDAC6 was measured (means ± SD, n = 3, *P < 0.05). **(D)** SNU-449 cells treated with tubacin for 6h and total cell lysates were subjected to western blot analysis as indicated. **(E, F)** SNU-387 and SNU-449 cells were treated with ACY-1215 for 24h and propidium iodide staining was used for cell cycle was analysis. **(G)** SNU-449 cells were treated with tubacin for 24h under hypoxia and the production of ATP was evaluated (means ± SD, n = 3, *P < 0.05). **(H)** Measurement of OCR in SNU-387 cells treated with Tubacin for 6h under hypoxic conditions.

Previous studies have indicated that HDAC6 was involved in mediating stress responses during hypoxia (20). We found that HDAC6 was primarily localized to the cytoplasm of HCC cells under normoxic conditions, while hypoxia promoted its perinuclear redistribution. Furthermore, deacetylase activity was significantly increased under 1% oxygen treatment (**Figures 2A, B**). Chloroquine (CQ) was used to determine whether the activated HDAC6 was caused by autophagy, and the results showed that HDAC6 activity was suppressed upon autophagy inhibition (**Figure 2C**). Acetylation levels of the key HDAC6 substrate α-tubulin was used to verify its enzyme activity. Tubacin was a highly selective HDAC6 inhibitor belonging to aliphatic-chain hydroxamate family. The results showed compared with normoxia, acetylated α-tubulin was decreased under hypoxia while HDAC6 inhibitor tubacin significantly up-regulated its acetylation (**Figure 2D**). In order to explore the role of HDAC6 inhibition in HCC cell proliferation caused by hypoxia, we investigated cell cycle and the results indicated that HDAC6 inhibition showed features of G2 arrest (**Figures 2E, F**). We further considered the effect of HDAC6 inhibition in mitochondrial function. The results suggested suppressed HDAC6 could rescue the decreased oxygen consumption and increased ATP production (**Figures 2G, H**). These results suggested that autophagy-induced HDAC6 activity during hypoxia contributed to decreased mitochondrial OXPHOS.

### Hypoxia Controlled β-Catenin Signaling in HCC Cells

**Figure 3:**
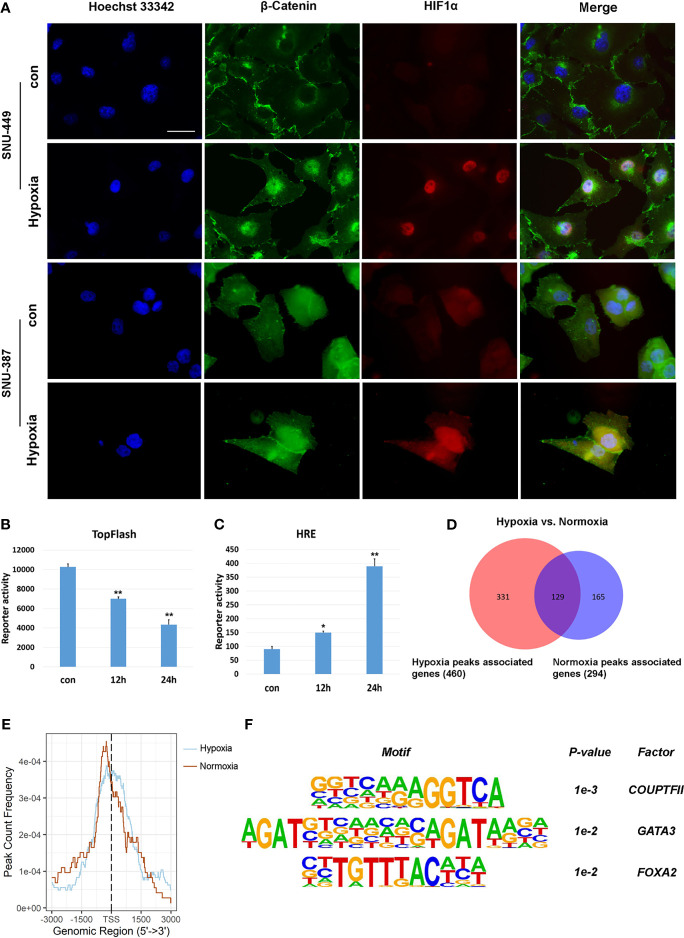
Hypoxia affected the transcriptional activation of β-Catenin signaling on target genes. **(A)** Immunofluorescence staining for β-Catenin in SNU-449 and SNU-387cells under normoxic or hypoxic conditions. Scale ba r= 60 μm. **(B, C)** TOP flash or HRE-promoter luciferase plasmid were transfected into SNU-387 cells (means ± SD, n = 3, *P < 0.05, **P < 0.01 *versus* the control group). **(D)** Venn diagram showing overlap between hypoxia group (red) and normoxia group (blue). **(E)** Analysis of hypoxia group and normoxia group ChIP peak signals. **(F)** Nucleotide binding motifs of β-Catenin under hypoxic conditions.

β-catenin functions as the core transcriptional coactivator of the canonical Wnt pathway and translocate from the cytoplasm to the nucleus, where it combines with TCF/LEF to promote target gene expression (21). Our results showed that hypoxia increased nuclear β-catenin levels in SNU-387 and SNU-449 cells (**Figure 3A**). However, HCC cells under hypoxia showed higher HIF1α transcription activity but lower TCF/LEF transcription activity (**Figures 3B, C**). To further investigate the role of nuclear β-catenin accumulation under hypoxia, chromatin immunoprecipitation with parallel DNA sequencing (ChIP-seq) was performed to identify potential target genes. In total, 625 β-catenin-enriched peaks were uncovered by ChIP-seq analysis. Among them, only 20.6% (129/625) were recovered in both the hypoxia and normoxia groups. Importantly, hypoxia induced differential expression of 71.9% (331/460) of these genes, suggesting that hypoxia may alter the transcriptional outputs of β-catenin (**Figure 3D**). Additionally, β-catenin binding was observed to be closer to transcription start sites during hypoxia, suggesting strong effects on gene expression under reduced oxygen (**Figure 3E**). Finally, we determined the diverse binding motifs between hypoxia and normoxia. The results suggested that COUP-TFII may be an important β-catenin cofactor under hypoxia (**Figure 3F**).

### HDAC6 Regulated the Interaction Between β-Catenin and COUP-TFII During Hypoxia

**Figure 4:**
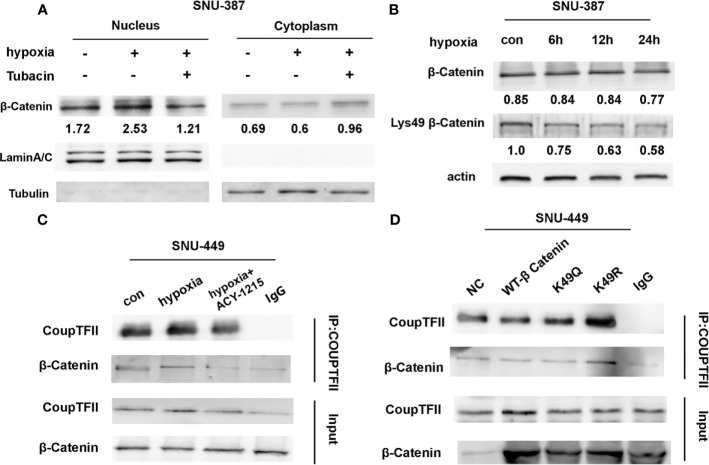
Acetylation affected the interaction between β-Catenin and COUP-TFII. **(A)** Western blotting was used to analysis the expression of β-Catenin in SNU-387 cells treated with Tubacin under hypoxia. **(B)** SNU-387 cells were exposed to hypoxic conditions and treated with Tubacin for 6h. Then total cell lysates were subjected to western blot analysis. **(C)** SNU-449 cells were exposed to hypoxic conditions and treated with ACY-1215 for 6h. Immunoprecipitation was performed using anti-COUP-TFII and precipitated proteins were identified by immunoblotting. **(D)** SNU-449 cells were transfected with wt-β-Catenin and β-Catenin acetylation-mimetic mutants and immunoprecipitation was performed as C described.

The affinity of β-catenin for a transcription cofactor such as TCF is regulated by lysine acetylation. We found that HDAC6 inhibition decreased the nuclear translocation of β-catenin that was induced by hypoxia (**Figure 4A**). The β-catenin N-terminus has acetylation sites that regulate its stability and signaling. Previous studies showed that Lys49 could increase TCF/LEF activity (22). Our results showed that Lys49 acetylation was suppressed during hypoxia (**Figure 4B**). ACY-1215 was another HDAC6 selective inhibitor belonging to aliphatic-chain hydroxamate derivative. More importantly, hypoxia promoted the interaction between β-catenin and COUP-TFII, while HDAC6 inhibition abolished this interaction (**Figure 4C**). To explore whether Lys49 site was involved in the interaction between β-catenin and COUP-TFII, we constructed the acetylation-mimetic mutant β-catenin-K49Q and deacetylation-mimetic mutant β-catenin-K49R. The results of expressing these mutant constructs in HCC cells suggested that Lys49 deacetylation could significantly enhance binding of β-catenin to COUP-TFII (**Figure 4D**).

### HDAC6 Inhibition Elevated Mitochondrial OXPHOS-Related Genes Regulated by COUP-TFII in HCC Cells

**Figure 5:**
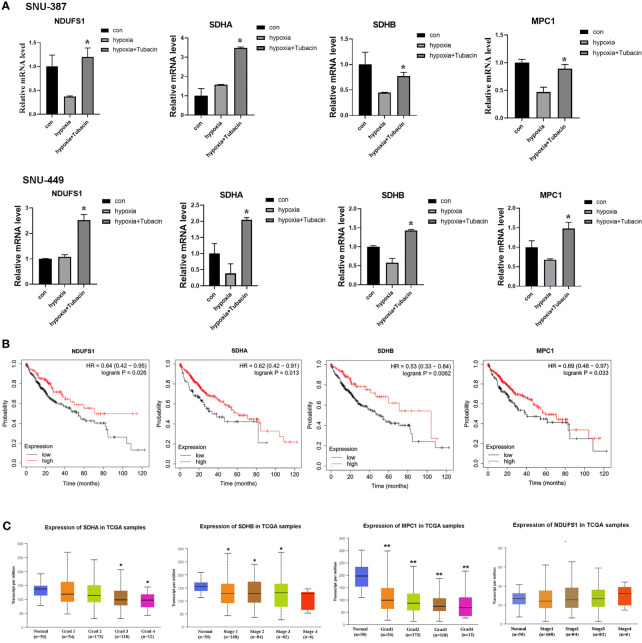
HDAC6 inhibition elevated mitochondrial OXPHOS-related genes regulated by COUP-TFII in HCC. **(A)** SNU-387 and SNU-449 cells were treated with Tubacin for 12h under hypoxia and mRNA level of NDUFS1, SDHA, SDHB and MPC1 were analyzed (means ± SD, n = 3, *P < 0.05 *versus* the hypoxia group). **(B)** Kaplan-Meier survival analysis was conducted to assess the NDUFS1, SDHA, SDHB and MPC1 expression in liver hepatocellular carcinoma. **(C)** Investigation of SDHA, SDHB MPC1 and NDUFS1 expression in different histologic grade of liver hepatocellular carcinoma, *P < 0.05, **P < 0.01 versus normal group.

Current studies indicated COUP-TFII was critical for transcription repression of mitochondrial electron transport chain enzyme, mitochondrial biosynthesis, mitochondrial pyruvate transport, oxidative stress detoxification and mitochondrial dynamics (23).In this study, we investigated the mRNA expression of genes encoding respiratory chain complex I, complex II and mitochondrial pyruvate carrier that reported regulated by COUP-TFII. The results indicated HDAC6 inhibition was effective to reverse these genes suppression caused by hypoxia in HCC cells (**Figure 5A**). Kaplan-Meier Plotter (http://kmplot.com/analysis/) online database was used for analyzing prognostic value of gene mRNA expression. 384 liver hepatocellular carcinoma patients were divided into high and low expression group based on auto selected performing threshold computed by the website. Hazard ratios (HRs) with 95% confidence intervals (CI) and log-rank P-values were calculated. UALCAN (http://ualcan.path.uab.edu) was used to analyze the association of mRNA expressions with clinicopathologic parameters. The results showed patients with SDHA, SDHB and MPC1 low expression may indicate more malignant type and shorter survival times (**Figures 5B, C**). These results suggested that HDAC6 inhibition may be a promising method for regulating mitochondrial OXPHOS during hypoxia through β-catenin/COUP-TFII axis.

### Targeting HDAC6 Suppressed HCC Growth *In Vivo*

**Figure 6:**
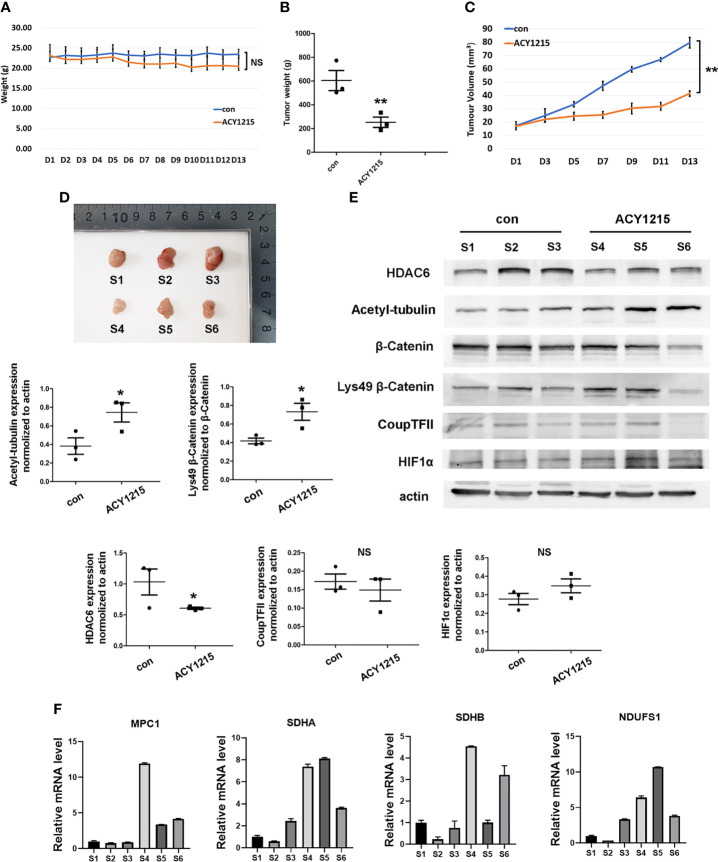
Inhibiting HDAC6/β-Catenin signal suppressed HCC growth *in vivo*. **(A)** Bodyweight was investigated each day. **(B)** Xenograft tumor weight was recorded on day 13 (means ± SD, n = 3, **P < 0.01). **(C)** Tumor volumes was determined by length and width measurement (means ± SD, n = 3, **P < 0.01). **(D)** Image of excised tumors from each groups. **(E)** Tumor tissues from mouse xenograft model were lysed in RIPA and total cell lysates were subjected to western blot analysis. S1-S3 were from control group and S4-S6 were treated with ACY-1215 (means ± SD, n = 3, *P < 0.05; ns, not significant). **(F)** Tumor tissues were lysed in TRIzol regent and mRNA level of NDUFS1, SDHA, SDHB and MPC1 were analyzed.

Currently, HDAC6 inhibitor ACY-1215 has been reported under clinical trials for different cancer therapy either monotherapy or in combination with other drugs, such as metastatic breast cancer and myeloid leukemia, which suggested targeting HDAC6 may become promising strategy in cancer therapy (12, 24). We next investigated the effects of using the HDAC6 inhibitor ACY-1215 *in vivo* on an HCC xenograft tumor model. Two-weeks after tumor inoculation, mice were divided into control and ACY-1215 treatment groups. The results showed that there was no significant change in body weight between the two groups (**Figure 6A**). Importantly, tumors treated with ACY-1215 grew slower than those in the control group (**Figures 6B**–**D**), indicating that HDAC6 inhibition was also effective for HCC *in vivo*. After investigating the expressions of the target proteins and genes in each sample, we found HDAC6 was down-regulated and acetylated α-tubulin was accumulated following ACY-1215 treatment. Furthermore, western blot analysis revealed improved levels of deacetylated β-catenin Lys49 in therapy group (**Figure 6E**). In addition, less expression of β-catenin deacetylation in sample 6 may blame on its low base level of β-catenin. Consistent with the experiments *in vitro*, the mRNA level of MPC1, SDHA, SDHB and NDUSF1 were increased with HDAC6 inhibition compared with control group (**Figure 6F**). These findings suggested that β-catenin/COUP-TFII signaling was involved in tumor suppression following ACY-1215 treatment *in vivo*.

## Discussion

**Figure 7:**
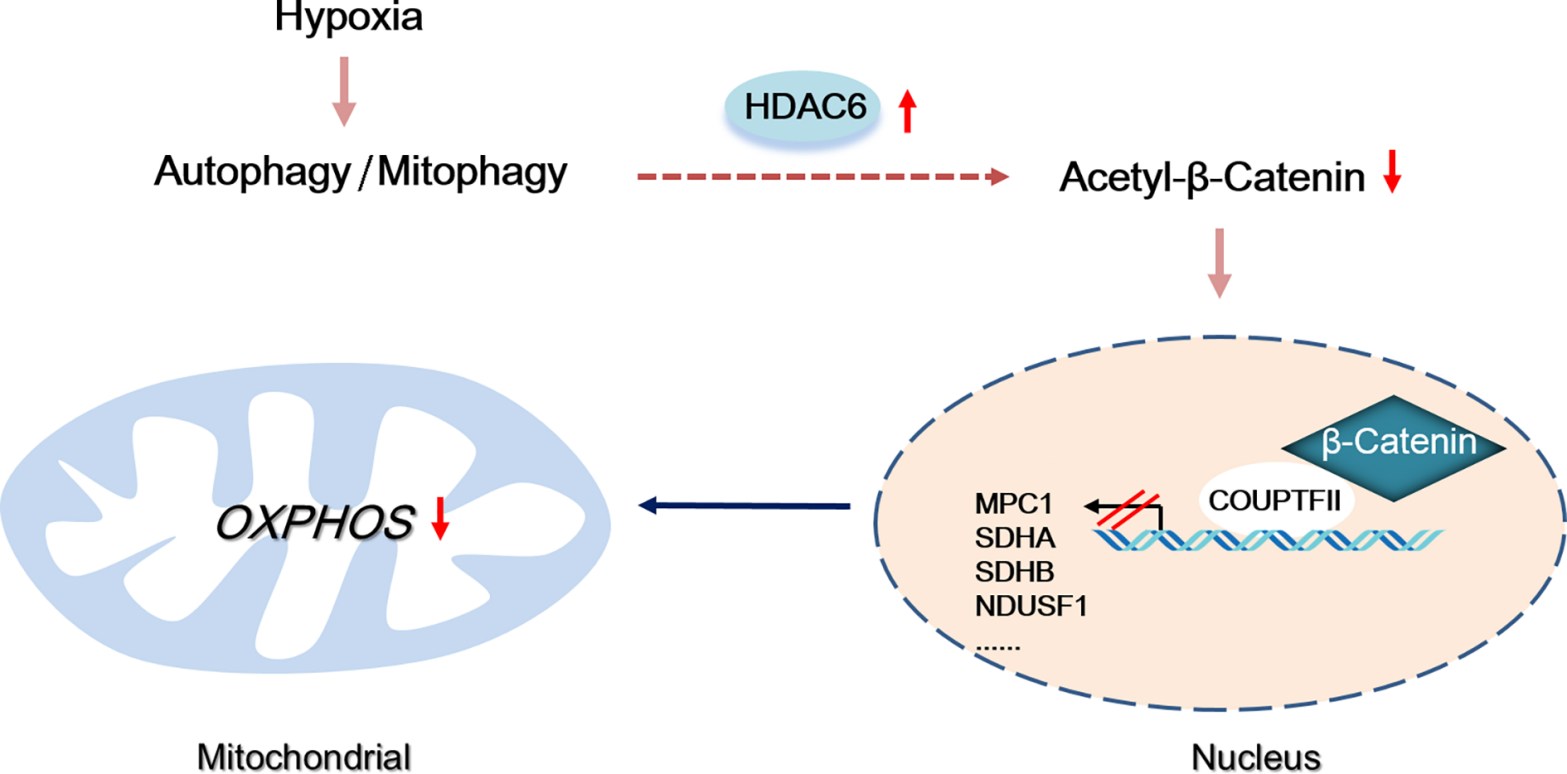
Proposed model by which autophagy-induced HDAC6 inhibited mitochondrial OXPHOS through regulating β-Catenin acetylation during hypoxia.

Multi cellular organisms have evolved mechanisms to rapidly adapt to hypoxia, and these pathways have been co-opted to prolong the survival of HCC cells. Coordination between mitochondria and the nucleus is necessary for maintaining the energy supply and inhibiting the toxicity induced by hypoxia (25). In this study, we found hypoxia induced-autophagy increased HDAC6 activity in HCC cells which was responsible for mitochondrial energy metabolism suppression. Our studies indicated HDAC6-mediated Lys49 deacetylation not only promoted β-catenin nuclear translocation but changed its binding transcription partners to COUP-TFII (**Figure 7**).

In our study, hypoxia caused autophagy on mitochondrial. The selective degradation of mitochondria through autophagy is called mitophagy Studies have suggested that hypoxia and/or low nutrient conditions induce metabolic stress that can trigger mitophagy (26, 27). Our data showed that under hypoxia, mitochondrial mass was significantly reduced, and autophagy-related proteins were recruited to mitochondria, indicating that hypoxia induced mitophagy in HCC cells. As shown in a previous study, the nuclear translocation of Nrf2 and TFEB are promoted by mitophagy (28); thus, we speculated that mitophagy is not only a catabolic process but is also involved in transcriptional responses *via* retrograde signaling. HDAC6 was discovered as a histone deacetylase; however, its role in regulating cytoplasmic protein acetylation has been revealed by subsequent studies. Currently, it is suggested that pharmacological inhibition of HDAC6 could improve mitochondrial fusion and motility (29). Other studies have indicated that HDAC6 regulates mitochondrial motility through the deacetylation of Miro1 (30). We found mitochondrial OXPHOS and ATP production were improved following HDAC6 pharmacological inhibition. Importantly, autophagy suppression through CQ could inhibit HDAC6 activity, which indicated that mitophagy may be involved in HDAC6 activation in HCC cells. However, the precise mechanism of mitophagy process induced by hypoxia is still unclear. Importantly, mitophagy pathway shares core molecular mechanisms with macroautophagy and they may both contribute to HDAC6 activation. Further study is needed to clarify how mitophagy regulates HDAC6 activity during hypoxia in HCC cells.

The nuclear translocation of β-catenin is key to activating the proliferative signaling mediated by TCF/LEF family members. Previous studies indicated under nutrient deprivation conditions, β-catenin formed complex with LC3 on the autophagosome membrane following degradation by autophagy (31). Studies on HepG2 and SMMC-7721 cells showed autophagy may inhibit the phosphorylation of β-Catenin at serine 33 and promote its nuclear translocation (32).Our experiments found hypoxia did not significantly affect the protein expression of β-catenin in HCC cells, but improved its nuclear expression. Moreover, inhibition HDAC6 activity could suppress its nuclear transport during hypoxia, which indicated that HDAC6-mediated β-catenin deacetylation resulted in its nuclear accumulation in HCC cells. Previously, it suggested the nuclear translocation of β-catenin results in binding to other transcription factors such as HIF-1α to initiate the transcription of glycolysis-promoting genes under hypoxic conditions (33). Consistent with these findings, our results indicated that although there was a large pool of nuclear β-catenin in HCC cells during hypoxia, there was reduced transcription activity of TCF/LEF. Furthermore, ChIP-seq was used to determine DNA sequences bound by β-catenin that were specific to hypoxic conditions. Motif analysis of the isolated DNA fragments suggested an association of β-catenin with the transcriptional repressor COUP-TFII, which we confirmed through co-immunoprecipitation assays. Importantly, HDAC6 inhibition could effectively block the interaction between COUP-TFII and β-catenin, which supported the conclusion that an acetylation modification was involved in regulating β-catenin signaling during hypoxia in HCC cells. We further investigated the specific acetylation site required for the interaction with COUP-TFII, which revealed that acetylation-deficient/mimetic mutants of β-catenin at Lys49 controlled the interaction with COUP-TFII.

Low oxygen levels result in the suppression of mitochondrial OXPHOS-related genes such as those involved in pyruvate metabolism and the electron transport chain, which induces a metabolic shift from mitochondrial OXPHOS to glycolysis (34). Mitochondrial import of pyruvate by the MPC complex is the first step of OXPHOS, which is regulated by oxygen concentrations (35). Suppressing COUP-TFII by RNAi upregulated MPC1 expression (36). Furthermore, current studies observed the negatively correlation between COUP-TFII and genes encoding mitochondrial electron transport chain in complex I, complex II and complex III, which suggested COUP-TFII may play pivotal roles in mitochondrial energy metabolism inhibition (37). In this study, we investigated the expression of MPC1 and the reported COUP-TFII correlated genes in electron transport chain *in vitro* and *in vivo*. The results confirmed inhibiting the HDAC6/β-catenin/COUP-TFII axis could increase their expression in HCC cells under hypoxia. However, SDHA in SNU-387 cells and NDUFS1 in SNU-449 cells were not significantly down-regulated under hypoxia. We speculated there may exist several compensatory response in their regulation such as reported sirtuin and the transcription factor MDM2 activation (38, 39). In addition, NDUFS1 currently didn’t show correlation with patients’ clinicopathologic parameters, which may be related to its low expression in liver hepatocellular carcinoma. Furthermore, the ChIP-seq also found other peaks related to mitochondrial energy metabolism such as ACSS1 (acyl-CoA synthetase short chain family member 1), CYP11A1 (cytochrome p450 family 11 subfamily A member 1) and mitochondrial fusion mediator MFN1 (mitofusin 1). The changes of these genes caused by β-Catenin deacetylation and their roles in regulating mitochondrial function during hypoxia deserve further attention. Briefly, we conclude that HDAC6-mediated β-catenin deacetylation may control mitochondrial OXPHOS by regulating the interaction between β-catenin and the transcriptional repressor COUP-TFII.

In summary, this study provided evidence that hypoxia-induced autophagy is a critical player in integrating mitochondrial energy metabolism with nuclear transcriptional responses. Autophagy activated HDAC6 regulates β-catenin acetylation and alters its binding to transcription cofactors in HCC cells. Targeting HDAC6 may become a novel strategy to disturb crosstalk between mitochondria and nucleus, which could inhibit HCC cell survival caused by hypoxic condition.

## Data Availability Statement

The datasets generated for this study were deposited in the NCBI Gene Expression Omnibus (GEO) database (GSE186801).

## Ethics Statement

The animal study was reviewed and approved by Ethics Committee of Jilin University School of Basic Medical Science.

## Author Contributions

JS and XY designed the study. XY, BL, and JW performed the experiments and drafted the manuscript. YZ and LX performed the *in vivo* xenograft experiments. XQ and SY interpreted the results. LW and JS edited and approved the manuscript. All authors contributed to the article and approved the submitted version.

## Funding

National Natural Science Foundation of China (81672948, 81772794, 82102733). Jilin Provincial Research Foundation for the Development of Science and Technology Projects (20190201164JC, 20200703009ZP, 20191008011TC). Jilin Provincial Finance for the Health Special Projects (2020SCZT099, 2021JC034, 2020Q010).

## Conflict of Interest

The authors declare that the research was conducted in the absence of any commercial or financial relationships that could be construed as a potential conflict of interest.

## Publisher’s Note

All claims expressed in this article are solely those of the authors and do not necessarily represent those of their affiliated organizations, or those of the publisher, the editors and the reviewers. Any product that may be evaluated in this article, or claim that may be made by its manufacturer, is not guaranteed or endorsed by the publisher.
